# Supplementary material for: Body Height of MPS I and II Patients after Hematopoietic Stem Cell Transplantation: The Impact of Dermatan Sulphate
Source: Diagnostics (Basel). 2024 Sep 4;14(17):1956. doi: 10.3390/diagnostics14171956 (PMC11394317; doi:10.3390/diagnostics14171956)
Supplement: Supplementary file 1 [file diagnostics-14-01956-s001.zip › diagnostics-3115989-supplementary.pdf]

Supplementary Figure S1. Individual patient's growth charts (green arrow- time of HSCT).

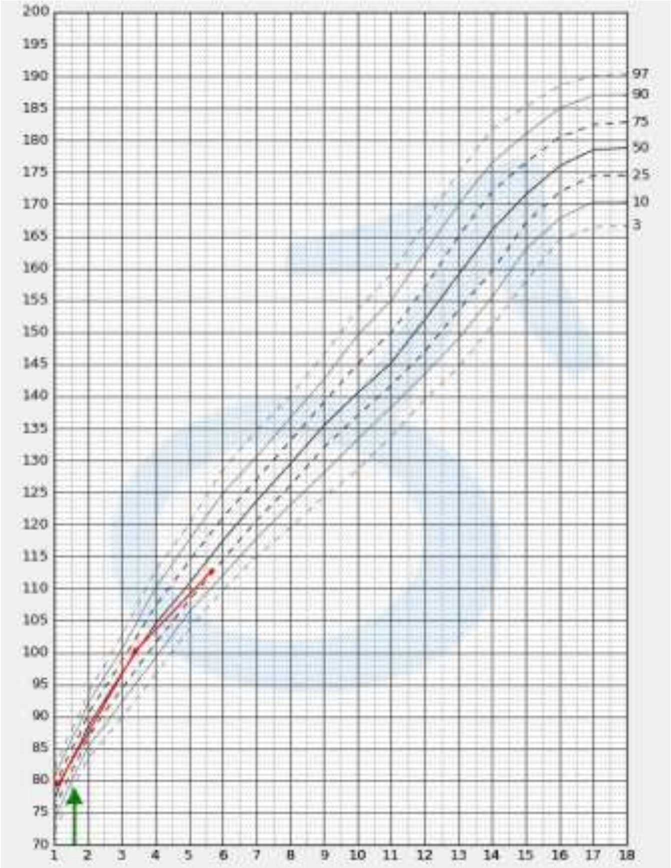

Patient 1

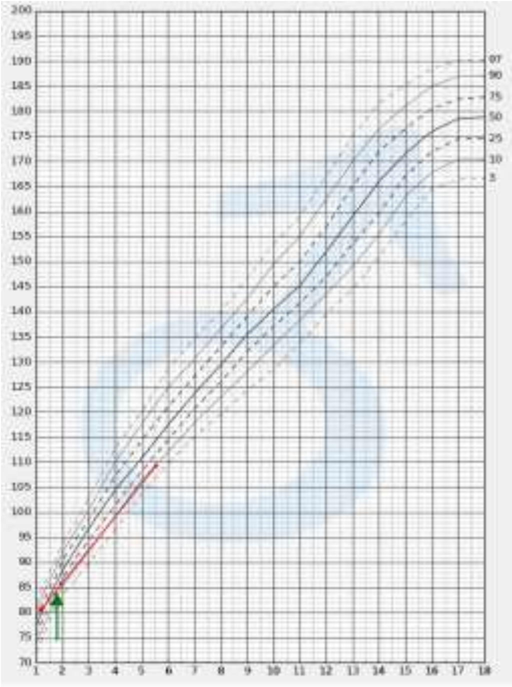

Patient 2

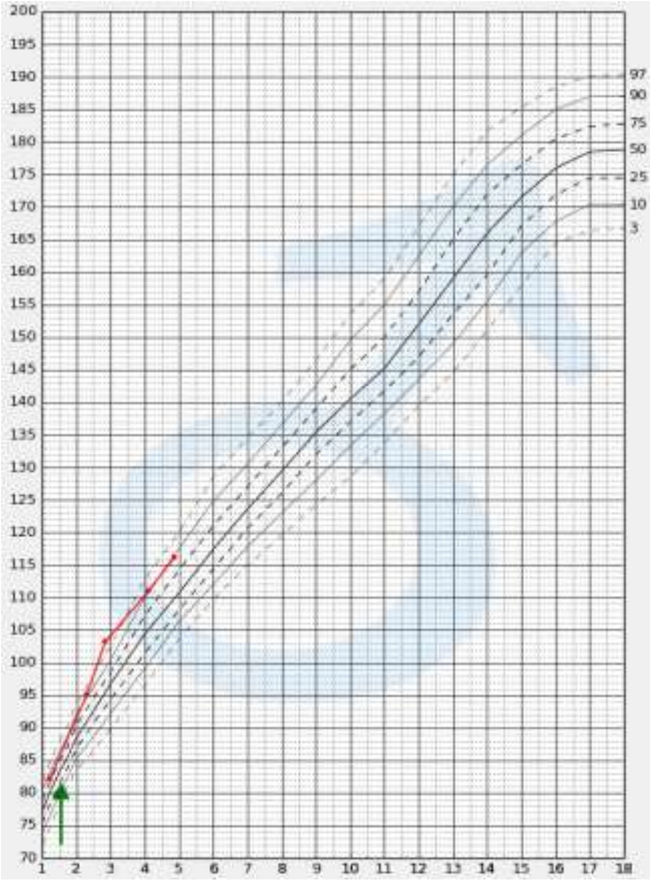

Patient 3

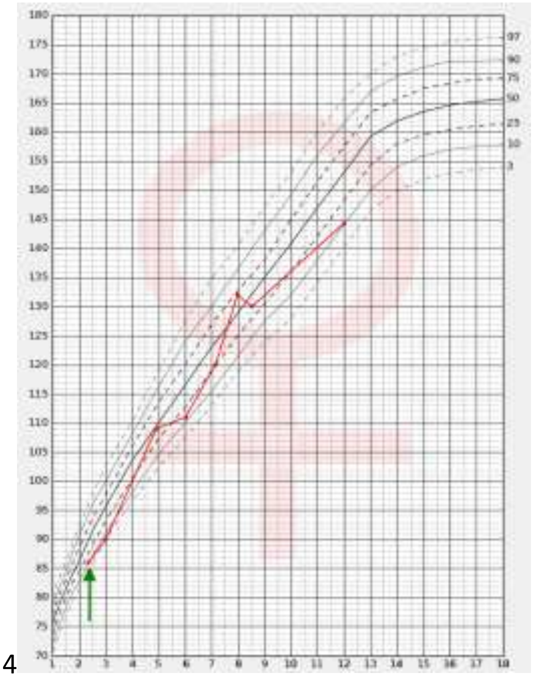

Patient 4

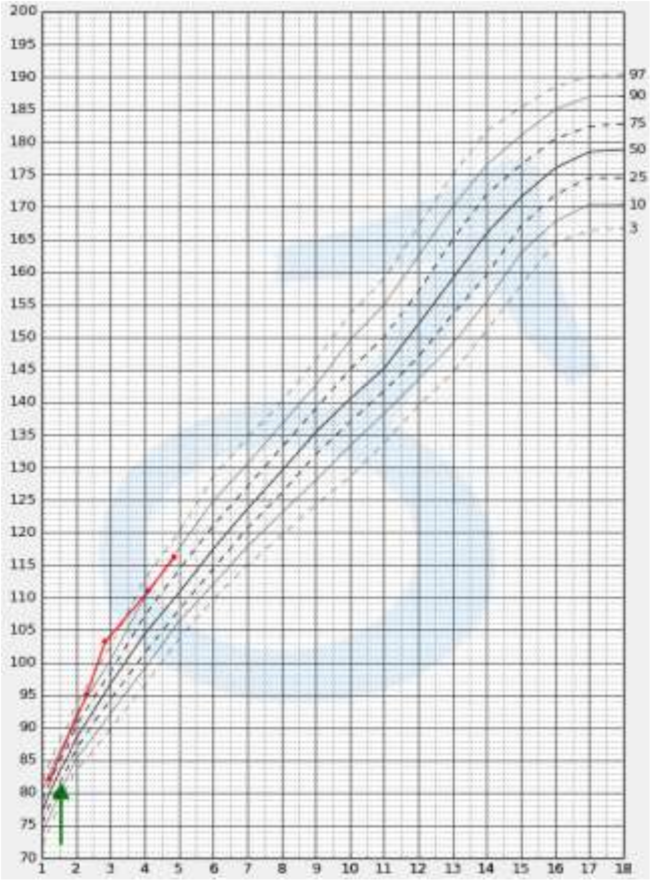

Patient 5

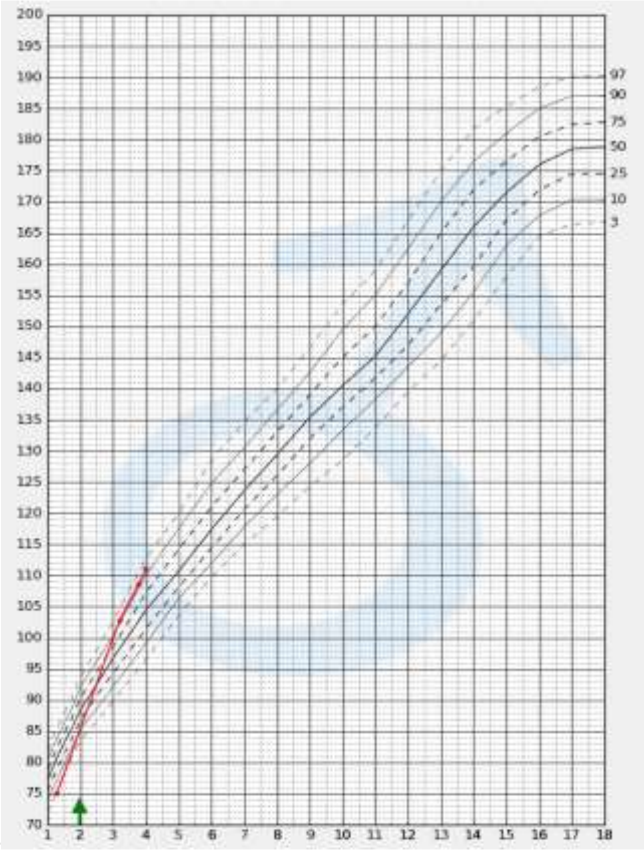

Patient 6
